# Correction: Diabetic retinopathy among type 2 diabetes mellitus patients in Sabah primary health clinics–*Addressing the underlying factors*

**DOI:** 10.1371/journal.pone.0264247

**Published:** 2022-02-15

**Authors:** Nurul Athirah Naserrudin, Mohammad Saffree Jeffree, Nirmal Kaur, Syed Sharizman Syed Abdul Rahim, Mohd Yusof Ibrahim

There is an error in the Corresponding Author designation. The correct Corresponding Author is Mohammad Saffree Jeffree, and the Corresponding Author’s correct email address is: saffree@ums.edu.my.

## References

[1] Naserrudin NA, Jeffree MS, Kaur N, Syed Abdul Rahim SS, Ibrahim MY (2022) Diabetic retinopathy among type 2 diabetes mellitus patients in Sabah primary health clinics–*Addressing the underlying factors*. PLoS ONE 17(1): e0261249. 10.1371/journal.pone.0261249 35089931 PMC8797256

