# Supplementary material for: Diabetic retinopathy among type 2 diabetes mellitus patients in Sabah primary health clinics–Addressing the underlying factors
Source: PLoS One. 2022 Jan 28;17(1):e0261249. doi: 10.1371/journal.pone.0261249 (PMC8797256; doi:10.1371/journal.pone.0261249)
Supplement: Attachment — Submitted filename: Response to Reviewers.docx [file pone.0261249.s001.docx]

Journal : Plos One

Manuscript Title : Diabetic Retinopathy Among Type 2 Diabetes Mellitus Patients in Sabah Primary Health Clinics – The Underlying Factors

Dear Editor and Reviewers.,

We would like to take this opportunity to thank Editor and All Reviewers for your time in reviewing this manuscript. All the comments and suggestions have helped to further improve this manuscript. We have thoroughly addressed all the comments and suggestions by the editor and reviewers. Please find below all the answers for your kind perusal.

Thank you.

Regards.,

Dr Athirah Naseer

**Journal Requirement**

| **No.** | **Journal’s Comments** | **Answer** | **Remark** |
| --- | --- | --- | --- |
| 1. | Please ensure that your manuscript meets PLOS ONE's style requirements, including those for file naming. The PLOS ONE style templates can be found at  <https://journals.plos.org/plosone/s/file?id=wjVg/PLOSOne_formatting_sample_main_body.pdf> and  https://journals.plos.org/plosone/s/file?id=ba62/PLOSOne_formatting_sample_title_authors_affiliations.pdf | Thank you for the reminder. We have carefully follow PLOS One style templates. |  |
| 2. | Please provide additional details regarding participant consent. In the ethics statement in the Methods and online submission information, please ensure that you have specified (1) whether consent was informed and (2) what type you obtained (for instance, written or verbal, and if verbal, how it was documented and witnessed). If your study included minors, state whether you obtained consent from parents or guardians. If the need for consent was waived by the ethics committee, please include this information.  If you are reporting a retrospective study of medical records or archived samples, please ensure that you have discussed whether all data were fully anonymized before you accessed them and/or whether the IRB or ethics committee waived the requirement for informed consent. If patients provided informed written consent to have data from their medical records used in research, please include this information.  Once you have amended this/these statement(s) in the Methods section of the manuscript, please add the same text to the “Ethics Statement” field of the submission form (via “Edit Submission”). | The need for consent was waived by the ethic committee. We have added the statement accordingly. Thank you | 109-111 |

**Reviewer 1**

| **No.** | **Reviewer’s Comments** | **Answer** | **Lines (according to the clean track)** |
| --- | --- | --- | --- |
| 1. | Are there any supporting information files? If yes, I don’t see them. | We are truly apologizes that we have clicked the wrong option. The data could not be shared publicly due to the confidential information of the patient. To access the data, an official letter to the Sabah Health State is required. We have changed the option accordingly. |  |
| 2. | Lines 97-103, the authors talk about the unique characteristics of the state of Sabah and how it may cause a difference in the prevalence of DR. However, this has not been discussed adequately throughout the rest of the manuscript. How do the findings support the low prevalence of DR in Sabah? What is the dependence of DR on these external factors? How can one address this issue? | The geographical difference between Sabah and other peninsular states in Malaysia relates to the limitation of healthcare facilities and equipment. This cause difficulties among the people to go and seek treatment which may relates to lower prevalence of DR in Sabah compared to other states. We have change the statement accordingly.  However, it’s worth nothing that this study only focused on the impact of gender, age, duration of T2DM diagnosis and health indices such as HBA1c, blood pressure lever, dyslipidaemia, VMI and comorbidities and complications such as nephropathy, cerebrovascular disease, ischemic heart disease, and DFU towards DR. The impact of geographical factor towards DR is not being explored in the data analysis. This is one of the important factor that requires further attention and has been highlighted as the study’s limitation. | 76-91  283-286 |
| 3. | There are a couple of other papers on the prevalence of DR in other regions of Malaysia that have not been addressed/included in the manuscript. I have included some of the article links below, however, the authors should look into more relevant papers and include them in the discussion of this manuscript.  <https://www.ncbi.nlm.nih.gov/pmc/articles/PMC7590874/> <https://pubmed.ncbi.nlm.nih.gov/25470640/> | Thank you for the suggestion. More Malaysian studies have been added in the manuscript. |  |
| 4. | The authors lightly touch upon proliferative and non-proliferative DR. Can the authors explain and address this in more detail? Also, what is the relevance of the type of DR with the main findings of this manuscript? | In the diabetes registry, DR is not categorized into either non-proliferative or proliferative. Thus the study highlighted the importance of this categorization. In non-proliferative it describes the early stage of DR - and the importance to intervene and improve diabetes control. We have add the explanation accordingly. | 50 - 55 |
| 5. | In Table 2, please define all the abbreviations in the column headings. Also, please write the full form of DFU in the table, so that the reader does not have to reference the text to understand the table data. | The table has been revised accordingly. |  |
| 6. | Line 100-102, what is the link between inadequate healthcare options for people of Sabah and the prevalence of DR? | This comment has been addressed together with comment number two. | 76-91 |
| 7. | In the methods, please have different sub-sections. | Sub-sections have been added according to the suggestion. | 99-132 |
| 8. | Tables 1 and 2, the authors may want to consider adding column borders to improve the clarity of the data. | The tables have been  revised accordingly. |  |
| 9. | In some places, the authors mention ‘ischaemic’, in other places it is ‘ischemic’. Please make it consistent. | Thank you for pointing this out. All the spelling have been standardized to ischaemic. |  |
| 10. | Lines 86-90, are very repetitive with the previous paragraphs. It seems redundant. The authors should concise the information presented. | The paragraph has been rearranged and rephrased accordingly. | 48-59 |
| 11. | Line 191 and 206, have spellings as HBA1c and HbA1c, respectively. Please make the capitalization of letters consistent throughout the manuscript. | All the spellings have been standardized to HbA1C. |  |
| 12. | Throughout the manuscript, there are inconsistencies with the abbreviation of Diabetes Mellitus. In some places it is mentioned as DM, in others T2DM. Kindly correct it. | All the abbreviations have been changed to T2DM. |  |
| 13 | Line 47, should be ‘this study attempts to…’ | This section has been omitted to follow the journal’s style and requirement |  |
| 14. | Line 48, should be ‘factors influencing DR…’ | This section has been omitted to follow the journal’s style and requirement |  |
| 15. | Line 54, should be ‘By understanding these underlying factors…’ | This section has been omitted to follow the journal’s style and requirement |  |
| 16. | Line 56, should be ‘this study could also become the benchmark…’ | This section has been omitted to follow the journal’s style and requirement |  |
| 17 | Line 62, should be ‘global incidence of DM in 2017 was as high as…’ | The phrase has been fixed accordingly. | 35 |
| 18 | Line 77, should be ’24 and 74 years old…’ | The phrase has been fixed accordingly. | 68 |
| 19 | Line 83, the word diagnosed is used twice in the same sentence. Please rephrase. | The phrase has been fixed accordingly. | 64-65 |
| 20. | Line 85, should be ‘develop some degree of…’ | The phrase has been fixed accordingly. | 66 |
| 21. | Line 93, requires a citation. | The citation has been added accordingly. | 54 |
| 22. | Line 104, it would be better to rephrase. | The sentence has been rephrased accordingly. | 92-93 |
| 23. | Line 143, should be ‘was 1.1 times higher than females…’ | The word “times” has been added accordingly. | 142 |
| 24. | Line 158, should be ‘risk factor were patients with a DFU…’ | The phrase has been edited accordingly. | 155 -157 |
| 25. | Line 160, should be ‘was 2 times higher than…’, in order to maintain consistency throughout the manuscript. | The phrase has been corrected accordingly. | 158 |
| 26. | Line 169, kindly rephrase to ‘from the findings of this study…’ | The phrase has been fixed accordingly. | 174 |
| 27. | Line 198, should be ‘other complications of diabetes…’ | The phrase has been fixed accordingly. | 14-215 |
| 28. | Line 229, needs a citation. | The reference has been added accordingly. | 253 |
| 29. | Line 242-243, should be one sentence instead of two. | Appropriate action has been taken. | 262-265 |
| 30. | Line 257-258, please make it concise, there are two ‘and’ in the sentence, which makes it redundant. | Appropriate action has been taken. | 275 - 276 |
| 31. | Line 268, should be ‘Acknowledgements’. | The letter “s” has been added accordingly. | 288 |

**Reviewer 2**

| No. | Reviewer’s Comments | Answer | Remark |
| --- | --- | --- | --- |
| 1. | Supplementary sheet to support data | We are truly apologizes that we have clicked the wrong option. The data could not be shared publicly due to the confidential information of the patient. To access the data, an official letter to the Sabah Health State is required. We have changed the option accordingly. |  |
| 2. | Mention about latest possibilities like pancreas transplantation and islet cell transplantation as an intervention (with latest references). It is expensive and puts financial burden on the community, so more important is good dietary habits and active lifestyle on a community level. | Appropriate action has be taken accordingly. | 183-215 |
| 3. | Overall need to address the latent phase of non-communicable disease like DM, HTN and modern interventions | This comment has been addressed according to the scope of the study. We only focused on the latent phase of DM and modern interventions. HTN is beyond the scope of the study. Thank you. | 211-215 |
| 4. | Title is clear. “ The Underlying Factors” needs to be rephrased to something like 'addressing the underlying factors'. | The title has been revised accordingly |  |
| 5. | Abstract: Conclusion. Page 2, line 37 “The effective management of the risk factors is crucial in preventing DR” can be changed to 'effective glycemic control.' | Appropriate action has been taken accordingly. | 28 |
| 6. | Main text: line 83: change 'diagnosis' to 'disease progression' 142: "the probability kindly re-phrase to 'The odds ratio was..' | Appropriate action has been taken accordingly. | 64 & 140 |
